# A tribute to professor Glyn Humphreys

**DOI:** 10.1016/j.neuropsychologia.2016.06.029

**Published:** 2016-11

**Authors:** Magdalena Chechlacz, Celine R. Gillebert

**Affiliations:** a Department of Experimental Psychology, University of Oxford, Oxford, UK; b Department of Brain and Cognition, University of Leuven, Leuven, Belgium

On January 14th we were deeply saddened to hear about the sudden death of Professor Glyn Humphreys. With this departure, the field of cognitive neuropsychology lost not only one of its most prominent and productive researchers, but also one of the most influential and beloved scientific mentors.

Glyn Humphreys was born on 28 December 1954 in Ormskirk, Lancashire. He obtained a Bachelor's degree in psychology in 1976, and graduated with a Ph.D in psychology in 1980, both from Bristol University, England. He then took up his first lectureship at Birkbeck College, London. Inspired by the scientific curiosity of his student, and future wife, Jane Riddoch, Glyn combined his passion for experimental psychology with the study of cognitive deficits in brain-injured patients. Glyn quickly appreciated that linking discrete patterns of brain damage to deficits in specific cognitive processes provides a powerful tool to test and inform theories about normal human cognition. He also rapidly became renowned for his research with brain-injured patients, not only because of the scientific merit of his work but also his unusual approach based on integrating patients into the life of his research group through numerous social events, including the famous Christmas parties. Over the years, numerous researchers visited Glyn to work with his panels of neuropsychological volunteers established first at the Brain and Behavioural Sciences Centre within the School of Psychology, University of Birmingham and later at the Cognitive Neuropsychology Centre, within the Department of Experimental Psychology, University of Oxford.

From the 1980s onwards, Glyn devoted himself with enthusiasm and thirst for knowledge to the field of cognitive neuropsychology. He made several conceptually important contributions to the understanding of cognitive and neural mechanisms of visual perception, attention, the control of action, memory, language and most recently social cognition. His work examining the mechanism of visual search and visual selection in different reference frames (e.g., spatial versus temporal search; viewer-centred versus object-centred coding) based on data from neuropsychological patients with neglect, extinction and simultanagnosia, provided an important theoretical framework underlying many papers published in this special issue, which he co-edited until his sudden death.

Glyn fully appreciated that in-depth study of a single patient with interesting and somewhat unusual cognitive problems can provide valuable insights into the functional architecture of the human brain. For instance, his studies in patients with Balint's syndrome (e.g., Humphreys and Riddoch, 2003, Cinel and Humphreys, 2006; Riddoch and Humphreys, 2004; Cooper and Humphreys, 2000) not only provided insights into the nature of deficits associated with this fascinating disorder in terms of interactions between object- and space based selection, but, importantly, challenged the traditional accounts of selective attention, which emphasized either object-based or space-based processes (following from the visual “what” and “where” dichotomy proposed by Ungerleider and Mishkin, 1982). The seminal paper “From What to Where: Neuropsychological Evidence for Implicit Interactions Between Object- and Space-Based Attention” presents a series of studies exploring coupling between object- and space-based attention in visual selection, cleverly demonstrating that spatial attention is directed towards the location of the winner from object-based selection rivalry (Humphreys and Riddoch, 2003). These experiments, most of them conducted on a single case with bilateral parietal damage, elegantly showed that the coupling between object- and space-based attention can function implicitly through early visual areas, despite the dysfunction of higher order areas (e.g., the parietal cortex) involved in the compilation of attention weights.

Throughout his career, Glyn's work remained at the forefront of both theoretical and methodological developments. He eagerly combined basic neuropsychological testing of brain-injured patients with functional neuroimaging studies and computational modelling. For instance, the selective attention and identification model (SAIM) of visual attention illustrates well how neuropsychological evaluation of attentional deficits in brain injured patients combined with brain activity data can influence computational modelling of visual attention. At the same time, the model also shows how computational models can provide theoretical accounts of the cognitive deficits reported in patients with brain damage (Heinke and Humphreys, 2003), in particular the underlying mechanisms of egocentric versus allocentric neglect.

Glyn's interests went beyond the field of cognitive neuropsychology, as he was eager to translate new knowledge to clinical applications. His joint passion for cognitive and clinical neuropsychology resulted in the development of two powerful assessment tools, the Birmingham Cognitive Screen (BCoS, Humphreys et al., 2012) and the Oxford Cognitive Screen (OCS, Demeyere et al., 2015), designed to diagnose in detail the cognitive problems resulting from stroke as well as to assist in targeting stroke rehabilitation. These neuropsychological test batteries are now used in more than 100 stroke units across the UK, and locally adapted versions are being tried out overseas, in several European countries, China and South Africa.

His achievements have been recognised by several awards: the Spearman Medal, the Prize for Cognitive Psychology (twice), the President's Award of the British Psychological Society, the Donald Broadbent Prize from the European Society for Cognitive Psychology, a Humboldt Fellowship, the Leibniz Professorship and Special Professorship of the Chinese Academy of Sciences. In 2015 the British Psychological Society honoured Glyn's exceptional talent and career in both research and education with the prestigious Lifetime Achievement Award.

More than anything else, Glyn transmitted his drive and passion to the next generations of neuropsychologists. Despite rapidly growing scientific prominence and the increasing amount of responsibilities associated with moving up through the academic ranks (Professor of Cognitive Neuropsychology and Head of Psychology, University of Birmingham and most recently the Watts Professor of Experimental Psychology and Head of Experimental Psychology, University of Oxford), he made his time available to his students and collaborators without any reservation. It is exactly this peculiarity that constitutes his biggest legacy. There is no doubt the field of neuropsychology lost an intellectual giant and an exceptional mentor. His sharp mind and attentive personality will be missed by hundreds of others. Glyn left behind a vast magnitude of theoretical and experimental work that will continue to inspire for many years to come.
